# Robotic infant surgery with 3 mm instruments: a study in piglets of less than 10 kg body weight

**DOI:** 10.1007/s11701-021-01229-0

**Published:** 2021-03-26

**Authors:** Thomas F. Krebs, Jan-Hendrik Egberts, Ulf Lorenzen, Martin F. Krause, Katja Reischig, Roberts Meiksans, Jonas Baastrup, Andreas Meinzer, Ibrahim Alkatout, Gesa Cohrs, Henning Wieker, Annette Lüthje, Sarah Vieten, Gerhard Schultheiss, Robert Bergholz

**Affiliations:** 1 Department of General-, Visceral-, Thoracic-, Transplant- and Pediatric Surgery, Faculty of Medicine, Christian-Albrechts-University of Kiel, UKSH University Hospital of Schleswig-Holstein Kiel Campus, Arnold-Heller-Strasse 3, 24105 Kiel, Germany; 2 Department of Pediatric Surgery, Children’s Hospital of East Switzerland, Claudiusstrasse 6, 9006 St. Gallen, Switzerland; 3 Department of Anesthesia and Intensive Care Medicine, UKSH University Hospital of Schleswig-Holstein Kiel Campus, Arnold-Heller-Strasse 3, 24105 Kiel, Germany; 4 Department of Pediatrics I and Pediatric Intensive Care Medicine, UKSH University Hospital of Schleswig-Holstein Kiel Campus, Arnold-Heller-Strasse 3, 24105 Kiel, Germany; 5 Department of Obstetrics and Gynecology, UKSH University Hospital of Schleswig-Holstein Kiel Campus, Arnold-Heller-Strasse 3, 24105 Kiel, Germany; 6 Department of Neurosurgery, UKSH University Hospital of Schleswig-Holstein Kiel Campus, Arnold-Heller-Strasse 3, 24105 Kiel, Germany; 7 Department of Cranio-Maxillo-Facial Surgery, UKSH University Hospital of Schleswig-Holstein Kiel Campus, Arnold-Heller-Strasse 3, 24105 Kiel, Germany; 8 Department of Animal Welfare, CAU Kiel, Olshausenstr. 40, 24098 Kiel, Germany

**Keywords:** Pediatric surgery, Robotics, Robotic surgery, Minimally invasive surgery

## Abstract

No data exist concerning the appication of a new robotic system with 3 mm instruments (Senhance®, Transenterix) in infants and small children. Therefore, the aim of this study was to test the system for its feasibility, performance and safety of robotic pediatric abdominal and thoracic surgery in piglets simulating infants with a body weight lower than 10 kg. 34 procedures (from explorative laparoscopy to thoracoscopic esophageal repair) were performed in 12 piglets with a median age of 23 (interquartile range: 12–28) days and a median body weight of 6.9 (6.1–7.3) kg. The Senhance® robotic system was used with 3 mm instruments, a 10 mm 3D 0° or 30° videoscope and advanced energy devices, the setup consisted of the master console and three separate arms. The amount, size, and position of the applied ports, their distance as well as the distance between the three operator arms of the robot, external and internal collisions, and complications of the procedures were recorded and analyzed. We were able to perform all planned surgical procedures with 3 mm robotic instruments in piglets with a median body weight of less than 7 kg. We encountered two non-robot associated complications (bleeding from the inferior caval and hepatic vein) which led to termination of the live procedures. Technical limitations were the reaction time and speed of robotic camera movement with eye tracking, the excessive bending of the 3 mm instruments and intermittent need of re-calibration of the fulcrum point. Robotic newborn and infant surgery appears technically feasible with the Senhance® system. Software adjustments for camera movement and sensitivity of the fulcrum point calibration algorithm to adjust for the increased compliance of the abdominal wall of infants, therefore reducing the bending of the instruments, need to be implemented by the manufacturer as a result of our study. To further evaluate the Senhance® system, prospective trials comparing it to open, laparoscopic and other robotic systems are needed.

## Introduction

Minimally invasive surgery (MIS) in children offers the ability to reduce incisional length, risk of infection, postoperative pain, and hospital stay [1]. Furthermore, operative and surgical precision appear to be improved with magnification of the intraoperative vision and the use of smaller size instruments than in open surgery—a benefit especially in pediatric patients [2]. Robotic, or computer assisted minimally invasive surgery may also be beneficial for pediatric surgical procedures, especially due to enhanced 3D Vision, stabilized camera, reduction of tremor, and downscaling of movements [3].

On the contrary, the diameter of the traditional robotic instruments (8 mm or 5 mm with longer articulating tails) and the consecutive distance between the ports needed for triangulation of the instruments towards the operative field limits the use of robotic systems in smaller children, such as infants and newborns[4–8].

With the introduction of 3 mm instruments into the portfolio of the Senhance® robotic system (Transenterix, USA) interventions on infants and newborns appear feasible [9, 10]. In a study applying inanimate models, the general application of this system, even in the smallest cavity with a volume as low as 92 ml, was feasible [11].

However, prior to any use in infants, it appears necessary to demonstrate the safety and practicability of specific pediatric surgical procedures in a live model. This has not happened so far. Thus, the aim of the study is to examine the feasibility and safety of pediatric surgical procedures with the Senhance® in an animal model simulating infants with a body weight lower than 10 kg.

## Materials and methods

### Surgeons

T.K., J.-H. E., and R.B. were registered as surgeons performing the procedures in this trial. T.K. and R.B. are pediatric laparoscopic and experimental fetal surgeons, J.-H. E. is a laparoscopic and robotic general adult surgeon and proctor for the Da Vinci robotic system. The procedures were performed by at least two of the three, acting as a team and switching from operating to assisting surgeon during the procedures as needed.

### Animals

This study was approved by the local animal rights and ethics committee (V242—13,326/2020, MELUND, Kiel, Germany). All animals used in the experimental laboratory were managed in compliance with federal and local laws for animal use and care, according to the ARRIVE guidelines and all institutional and national guidelines for the care and use of laboratory animals were followed [12]. 12 piglets with a weight below 10 kg were used. A day before the procedure, the animals were kept in three isolated groups to adapt to their surroundings.

### Anesthesia

Premedication of the piglets was initiated with midazolam (0.5 mg/kg), ketamine (25 mg/kg), and atropine (0.04 mg/kg) intraperitoneally. Anesthesia was induced with a propofol bolus dose (5–10 mg/kg intravenously) and maintained with a continuous infusion of propofol (5–10 mg/kg/h) given via an ear vein. After endotracheal intubation during spontaneous respiration, the animals were ventilated pressure-controlled with 30% oxygen and 14–29 breaths/min. The inspiratory time (Tinsp) was 1.0 s (0.7–2.0 s), the positive endexspiratory pressure (PEEP) was adjusted to 3–5 cm H2O and the inspiratory pressure (Pinsp) was focused up to 20 cm H2O. Ventilation was performed with the Draeger Primus (Dräger, Germany) and monitored oxygen and end-tidal carbon dioxide. Oxygen saturation (SpO2) was monitored by a continuous pulse oximeter (Carescape B650, GE, Solingen, Germany) placed on the animals tail. Meloxicam was given intra-muscularly (0.4 mg/kg) and metamizole 50 mg/kg intravenously every 4 h for analgesia. Vecuronium (0.2 mg/kg) was given as needed. Depth of anesthesia was judged according to heart rate and respirations as well as reaction to stimuli. If clinical assessment suggested a decreasing level of anesthesia, additional propofol and ketamine were injected.

At the end of the procedures, euthanasia was performed by intravenous administration of potassium-chloride (10 mol). Animals were observed 20 min to verify their death.

### Robotic system and instruments

The Senhance® robotic system, the 3 and 5 mm instruments and advanced energy devices were supplied by Transenterix as a research grant for R.B.

**Fig. 1:**
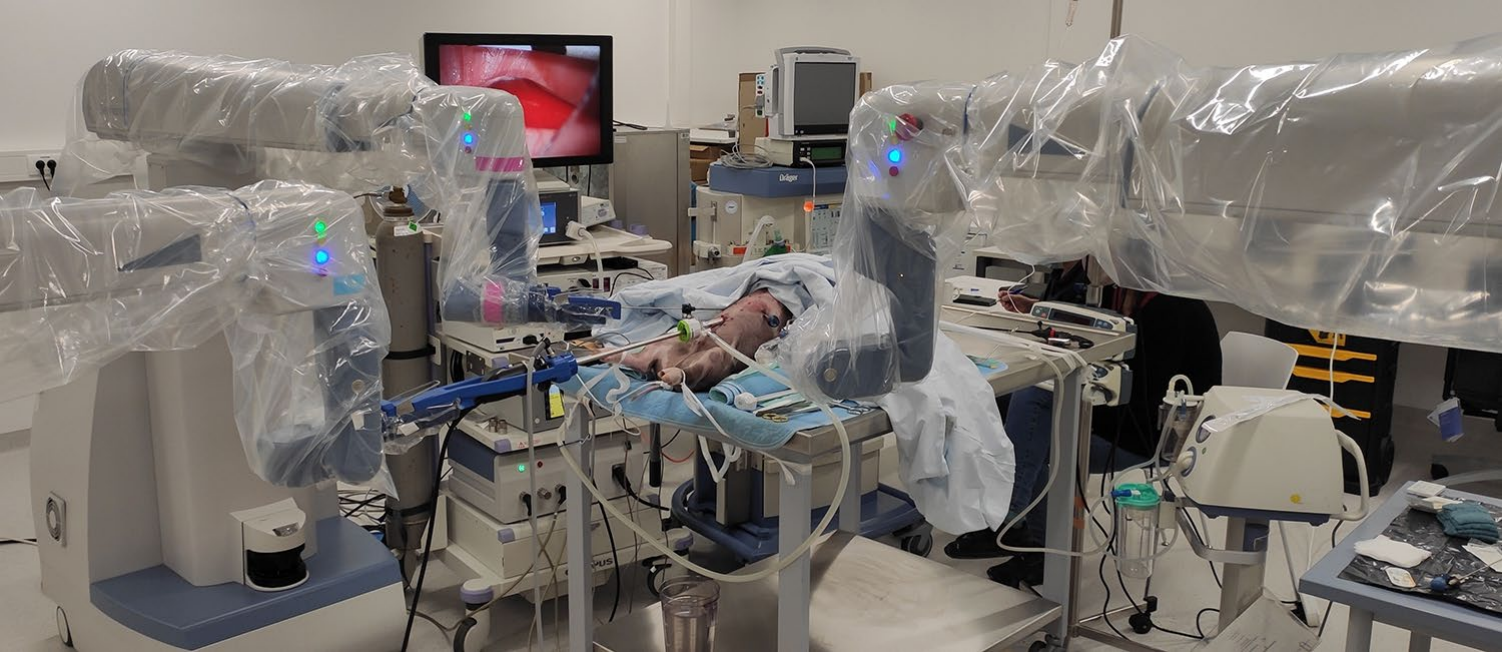
Setup of the experiments. The Senhance can be seen with its three arms, operating a 10 mm camera and a 3 mm instrument in the right and left hand arm. An accessory video screen for the 2D display of the operating field is on the left side. In the background, the Draeger Primus is positioned for anesthesia

The setup consisted of the master console and three separate arms, one with a 10 mm 3D 0° or 30° videoscope (Fig. 1). The instruments were applied as deemed necessary by the operating surgeons. For the procedures, the system was covered in sterile drapes and connected to a video system with an additional external monitor and recording capabilities (Sony, Germany). CO_2_-Insufflation was applied with the Endoflator® (Karl Storz, Tuttlingen), suction and irrigation were generated with the Unimat30® (Karl Storz, Tuttlingen).

Unless mentioned otherwise, all instruments used were of 3 mm diameter. The clip applier was adapted for the robotic arm (Hem-o-Lok®, Weck, USA). For internal retraction, a blunt grasper or 5 mm endoscopic fan retractor was used (ENDO-Retract 2, Covidien, Germany). The sutures used were Vicryl (Johnson and Johnson, Germany). All sutures were tied with intracorporal knots, as we deemed the robotic system being especially beneficial in laparoscopic intracorporal knot tying.

### Surgical procedures

All procedures were performed under general anesthesia in intubated and ventilated animals. The animals were placed in the supine or prone position, the abdomen or thorax were washed, shaved and disinfected. The robotic arms of the Senhance® were set according to the anticipated procedure and the sterile field and robot were draped.

The first procedure evaluated was the calibration of the system for calculating the fulcrum point: To minimize the force to the tissue at the trocar site the Senhance® system is measuring forces at the instrument shaft and calculates the fulcrum point with the lowest forces to the abdominal wall. For this calculation, the inserted instrument is carefully moved downwards. The results of these measurements are depending on the port the instrument is inserted through, the depth of the insertion of the port and instrument rigidity as well as tissue elasticity. It can be hypothesized that in newborns, with a much higher compliance or laxity of the abdominal wall, the automatic fulcrum point setting will depress the instruments too much before gaining enough force feedback for the calibration, not concluding the appropriate fulcrum point or even damaging the child.

Fulcrum point calibration was performed with all used instruments on every arm of the system. Additionally, at the start of the study, a specific setup with a three-port approach with different insufflation pressures ranging from 2 to 16 mm Hg and applying different 3 and 5 mm instruments and 10 mm videoscopes was used to evaluate the fulcrum point calibration for laparoscopy and thoracoscopy in the animals. Any collision or bending of the instruments as well as excessive depression with deformation of the body wall was recorded.

The further procedures we selected for evaluation were held as being the most common minimally invasive pediatric procedures. In case of unforeseen complications, the live procedure was terminated and completed in the sacrificed animal. Conversion to open or laparoscopic surgery for the management of complications was not included in this study.

The procedures selected were: fulcrum point calibration, explorative laparoscopy, clip ligation of the umbilical vein, cystocutaneostomy and cystostomy closure, cholecystectomy, cholecystoenterostomy, choledocho-enterostomy (biliodigestive anastomosis) or hepato-porto-enterostomy (Kasai procedure), esophageal resection and anastomosis, lobectomy of the right upper lobe, Nissen fundoplication, hiatoplasty, diaphragmatic plication, gastrostomy and gastrostomy closure, gastroenterostomy, gastro-gastrostomy, entero-enterostomies, atypical liver resection, nephroureterectomy, ureteroenterostomy, and pyeloplasty.

### Evaluation of the surgical procedures

In all procedures, the amount, size, and position of the applied ports, their distance (in cm, ΔLC: distance of the left hand instrument to camera, ΔRC, and ΔLR, respectively, ΔALC: distance between auxiliary left hand to camera port, ΔARC for the auxiliary right port) as well as the distance between the three operator arms of the robot (ΔARM-LC, ΔARM-RC, and ΔARM-LR) were recorded [11]. The abdominal dimensions of the piglets were recorded as abdominal length (LENGTH: distance between the xiphoid process and the pubic tubercle in centimeters) and abdominal width (WIDTH: maximum distance between the left and right abdominal wall in the supine position).

All procedures were video-recorded for later blinded analysis. Outcome parameters are: completion of the task (yes, no), amount of external instrument–instrument collisions (n) and amount of instrument—organ collisions (*n*).

Rational data were given as median and interquartile range (IQR). Due to the pilot character of this study, no comparison or statistical analyses were performed.

During the course of the study, we held daily evening video conferences with the staff of Transenterix to discuss technical issues and the progress of the study.

## Results

12 piglets with an age of 23 (12–28) days and a body weight of 6.9 kg (6.1–7.3 kg) were used. The duration of all procedures per piglet was 7 (6–11) hours. Their abdominal length was 22.5 (22–23.5) cm, the abdominal width was 14.5 (14.5–15) cm (Table 1).

**Table 1:** Animals used and procedures performed per animal

Procedure	Procedure	Animal	Pressure	ΔLC	ΔRC	ΔRL	ΔALC	ΔARC	Calibration	Complications	External collisions	Instrument organ collisions	Excessive bending of instruments
No		No	mmHg	cm	cm	cm	cm	cm	Camera, right or left port				
1	Fulcrum point calibration for laparoscopy	1	2–16	7.5	7.5	12	5		Camera: 2 fails (with pressures of 2 mmHg); instrument arms: 3 fails (with 10 cm insertion depth none)		0	0	0
2	Explorative laparocopy		6	7.5	7.5	12	5		3 fails (with 10 cm insertion depth none)		0	0	0
3	Cystocutaneostomy		4	7.5	7.5	12	5		3 fails (with 10 cm insertion depth none)		0	0	0
4	Cystostomy closure		4	7.5	7.5	12	5		3 fails (with 10 cm insertion depth none)		0	0	0
5	Cholecystectomy		5	5	8.2	16.1	5.1				0	0	0
6	Cholecystectomy	2	10	7	9.5	12					0	1	0
7	Esophageal resection and anastomosis		2–10	4,5	4	10					1	0	2
8	Nissen fundoplication	3	5–10	7	4	10	10.5	3.5			0	3	0
9	Gastrotomy and gastrostomy closure		5–10	7	4	10	10.5	3.5			0	0	0
10	Esophageal resection and anastomosis		5–10	5	4.5	11			Maximal insertion depth 2 cm with 3 fails at calibration of the camera and instruments		0	0	2
11	Esophageal resection and anastomosis	4	2–10	4,5	7	10			maximal insertion depth 2 cm with 3 fails at calibration of the camera and instruments		1	0	2
12	Gastro-gastrostomy		5–10	6,5	7.5	9.5	10.5	4			0	0	0
13	Atypical liver resection		5–10	6,5	7.5	9.5	10.5	4			0	0	0
14	Nissen fundoplication	5	8	7,5	8.5	10.5	9		1 fail (bipolar grasper, 1 cm insertion depth), success with deeper insertion		0	3	0
15	Gastroenterostomy		8	7,5	8.5	10.5					0	0	0
16	Diaphragmatic plication (left)		5–10	6	8	9.5		10			0	0	0
17	Nissen fundoplication	6	5–10	7	8	10				Perforation of caval vein	0	2	0
18	Hiatoplasty (dorsal and anterior)		5–10	7	8	10		12			0	3	0
19	Nephroureterectomy		5–10	5,5	6	10.5		12			2	1	1
20	Umbilical vein ligation (5 mm hemolock)	7	8	7,5	8	10.5			Left port: 1 fail (3 mm bipolar); right port: 2 fails (3 mm monopolar hook in 5 mm port)		0	0	0
21	Cholecystoenterostomy (biliary diversion)		5–10	7,5	8	10.5		9.5			0	1	0
22	Nephroureterectomy		5–10	7	5.5	10.5		12			0	1	0
23	Nissen fundoplication		5–10	6	5.5	11	6.5				0	2	0
24	Ureteroenterostomy	8	10	8,5	6.5	12			1 fail ( 3 mm bipolar)		0	1	0
25	Nephroureterectomy		10	8,5	6.5	12			1 fail ( 3 mm bipolar)	Perforation of the renal vein	0	1	0
26	Esophageal resection and anastomosis	9	4–8	5,5	6	11.5					0	0	1
27	Fulcrum point calibration for thoracoscopy		2–16	5,5	6	11.5					0	0	0
28	Pyeloplasty	10	5–10	5,5	6	10.5		12			0	0	0
29	Nephroureterectomy		5–10	5,5	6	10.5		12			0	2	0
30	Entero-enterostomy, running suture		5–10	6	7.5	9.5					0	0	0
31	Entero-enterostomy, interrupted suture		5–10	6	7.5	9.5					0	0	0
32	Nissen fundoplication		5–10	6,5	7.5	9.5	10	7			0	3	0
33	Lobectomy of the right upper lobe	11	5–10	4	6	9					0	2	2
34	Choledocho-enterostomy (Kasai hepatoportoenterostomy)	12	5–10	5	8.2	12		10			0	1	0

*ΔLC* distance of the left instrument to camera, *ΔRC* distance of the right instrument to camera, *ΔLR* distance of the left instrument to the right instrument, *ΔALC* distance between auxiliary left and camera port, and *ΔARC* distance between auxiliary right and camera port

Cumulatively over all procedures performed, the distance between the left hand and camera port was 6.5 (5.5–7.5) cm with a minimum distance of 4.5 cm for thoracoscopic esophageal resection and anastomosis. The distance between the right hand and camera port was 7.5 (6–8) cm with a minimum distance of 4 cm for Nissen fundoplication, gastrostomy, and gastrostomy closure. The distance between the left and right hand ports was 10.5 (10–12) cm, with a minimum distance of 9.5 cm during atypical liver resection and gastro-gastrostomy. Into the auxiliary right and left hand ports laparoscopic instruments were inserted for assistance, their distance to the camera port can be found in Table 1.

The distance between the base of the left hand robotic arm and the base of the camera arm was 1.57 (1.55–1.57) m, the distance between the base of the right hand robotic arm and the base of the camera arm was 1.5 (1.5–1.5) m, and between the left and right arm robotic base 1.97 (1.72–2) m, respectively.

### Fulcrum point calibration for laparoscopy and thoracoscopy

Calibration for the fulcrum point was successful in most of the procedures. The flexible 3 mm instruments as the Maryland grasper, monopolar hook, and 3 mm bipolar clamp and grasper were most prone for failed calibration which needed up to three repetitions and repositioning of the instruments. Excessive bending of the instruments was noted during calibration of the fulcrum point and during the procedures. Due to space restrictions, especially in the thorax, most instruments could not be inserted deeper than 1 cm before impending instrument—organ collision. The low insertion depth was found as a contributing factor to miscalculations of the fulcrum point. After tests with variable insertion depths, an insertion of at least 5–10 cm of the instrument resulted in reliable calibration but was often not accomplished due to the narrow restrictions of the operative space in piglets.

### Explorative laparoscopy

After insertion of the videoscope and two 3 mm grasping instruments, inspection and evaluation of the four abdominal quadrants was performed. Handling of and running the bowel could be accomplished with the three-port approach. Changing the working spaces from one quadrant to the horizontally adjacent could be performed without manually repositioning the arms and port placements. Changing working spaces and quadrants in a vertical manner required manually repositioning the videoscope and arms with new calibration.

### Clip ligation of the umbilical vein

This procedure was chosen as simple robotic dissection to begin with. The umbilical vein is easily approachable within the epigastrium. The piglet was placed in the anti-Trendelenburg’s position, the camera port was placed 3 cm below the umbilicus and the left and right robotic arm in triangulation to the umbilical vein.

The umbilical vein was fixed with a grasper in the left arm and a robotic 5 mm clip applier (Hem-o-Lok®) was inserted. Application of three clips and consecutive cutting the vein between the distal one and proximal two clips completed the procedure.

### Cystocutaneostomy and cystostomy closure

The piglet was placed in the Trendelenburg’s position, the camera port was placed in the umbilicus and the left and right 3 mm robotic arm in triangulation to the dome of the bladder. The dome of the bladder was identified and opened with monopolar hook cautery. The opening was sutured to a corresponding incision in the abdominal wall with interrupted 10 cm 3–0 Vicryl SH sutures. Then, the sutures of the cystocutaneostomy were taken down and the defect in the bladder closed with interrupted 10 cm 3–0 Vicryl SH sutures.

### Cholecystectomy (2 procedures)

The piglet was placed into anti-Trendeleburg’s position and slightly turned to its left side. The three robotic ports were placed with triangulation into the direction of the gallbladder with the camera port in the umbilicus. Due to the anatomy of the porcine liver, a fourth port was needed for liver retraction with a 3 mm blunt grasper. The neonatal piglet liver, as well as the gallbladder, is very sensitive to mechanical stress and forced grasping resulted in haemorrhage. After elevation of the liver and retraction of the gallbladder the infundibulum, the cystic duct and the cystic artery were identified. Ligation of the cystic artery and duct was accomplished with 4–0 Vicryl RB-1 or the 5 mm robotic clip applier (Hem-o-Lok®). Gallbladder dissection from the liver was performed with monopolar hook cautery. During the first procedure of the two, a bleeding from the cystic artery was controlled by clips.

### Cholecystoenterostomy

This procedure was chosen in the context of biliary diversion for biliary obstruction or treatment of progressive familial intrahepatic cholestasis. The piglet was placed into anti-Trendeleburg’s position and turned to its left side. The three robotic ports were placed with triangulation into the direction of the gallbladder with the camera port in the umbilicus. Due to the anatomy of the porcine liver, a fourth port was needed in the left upper abdomen for liver retraction with a 3 mm blunt grasper or 5 mm fan retractor (ENDO-Retract 2, Covidien, Germany).

**Fig. 2:**
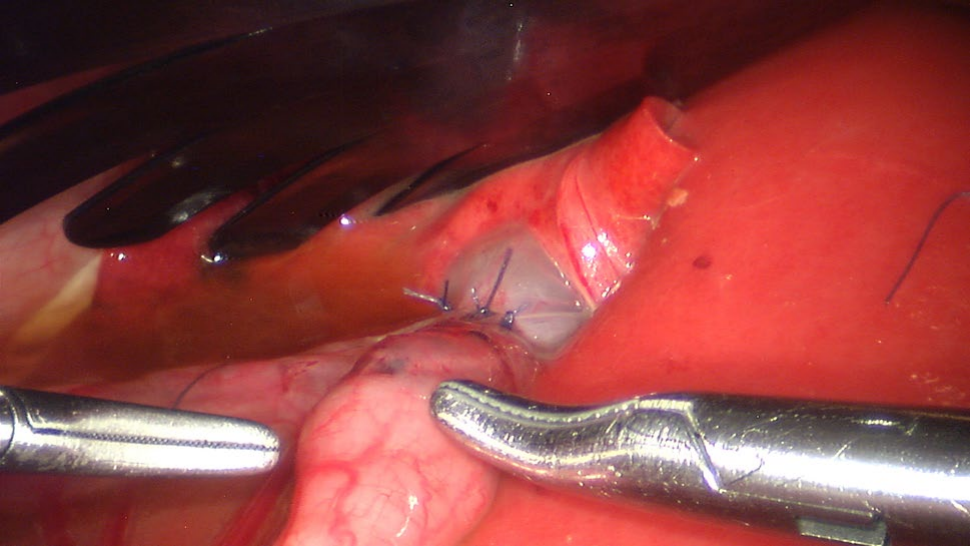
Cholecysto-enterostomy: this figure displays the small workspace the anastomosis was created in. To the left, the tip of a 3 mm Maryland grasper and to the right, a 5 mm needledriver can be seen, demonstrating the confined space in comparison to the instruments created by the fan retractor seen above. The anastomosis could be fashioned with interrupted Vicryl 5–0 TF-1 sutures

As the gallbladder is situated below the multiple lobes of the vulnerable right liver, retraction for anastomosis was intricate (Fig. 2). A 2 mm incision was set in the fundus of the gallbladder with 3 mm monopolar hook cautery. An adjacent loop of small bowel was opened longitudinally with cautery to a corresponding length. The omega shaped anastomosis was performed with interrupted sutures, the dorsal knots to the inside, ventrally to the outside (5–0 Vicryl TF-1).

### Choledocho-enterostomy (biliodigestive anastomosis), hepato-porto-enterostomy (Kasai procedure)

Both procedures were held to be difficult laparoscopic procedures worth evaluating with the robotic system.

As the liver of the piglets is very vulnerable, dissection into the porta hepatis for the Kasai procedure resulted in diffuse bleeding and loss of vision. Therefore, a choledocho-enterostomy as biliodigestive anastomosis was performed.

The piglet was placed into anti-Trendeleburg’s position and turned to its left side. The three robotic ports were placed with triangulation into the direction of the gallbladder with the camera port in the umbilicus. Due to the anatomy of the porcine liver, a fourth port was needed in the left upper abdomen for liver retraction with a blunt grasper or 5 mm fan retractor. The liver lobes above the gallbladder were retracted, the cystic artery was identified and suture ligated (5–0 Vicryl SH). The cystic duct was identified and separated, it was left open for further dissection towards the common bile duct. The gallbladder was removed from the liver and placed into the pelvis. The common bile duct was dissected just proximal to the liver. A loop of small bowel was identified and separated. A hockey stick-shaped anastomosis end to side of the hepatic duct to the small intestinal loop was created by 6–0 Vicryl TF-1 interrupted sutures. The footpoint anastomosis was fashioned as an end to side entero-enterostomy (5–0 Vicryl TF-1).

### Esophageal resection and anastomosis (4 procedures)

**Fig. 3:**
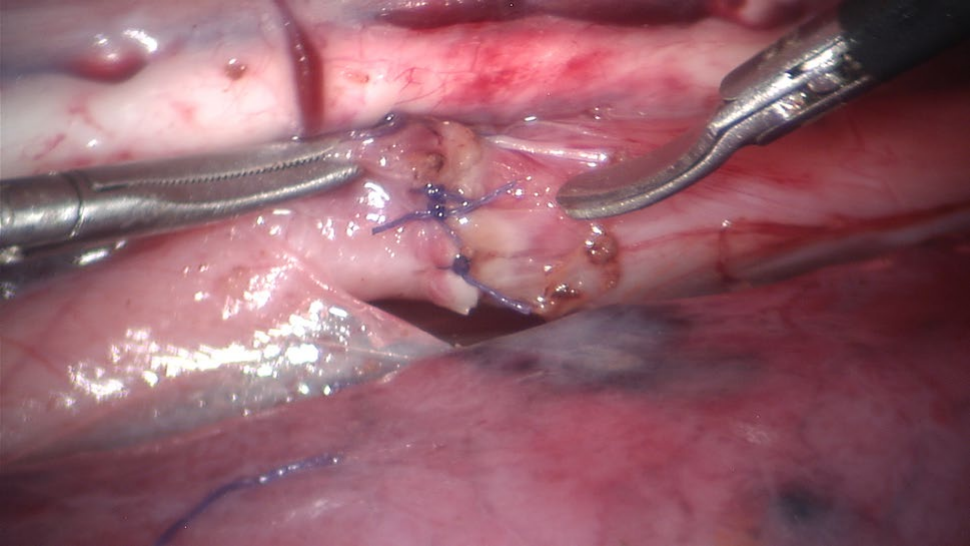
Esophageal reconstruction: this figure shows a 3 mm Maryland grasper and 3 mm scissors with a completed esophageal anastomosis (Vicryl 5–0 TF-1) in between

As we see the benefit of robotic assisted laparoscopy in complex procedures in small cavities, esophageal resection and anastomosis was a procedure we put emphasis on: access to the right thorax was gained with the lung compressed by insufflation of CO2 with a pressure starting with 4 mmHg. The piglet was ventilated on both sides and placed almost prone, the camera port was placed about 2 cm paraspinally to the right in extension of the right eye in the mid thoracic height. The ports for the 3 mm left and right instruments were placed cranially and caudally to the camera port and more anterior situated for triangulation to the esophagus. Access to the porcine thorax was possible, although the narrow intercostal spaces hampered calibration of the fulcrum point and led to excessive bending of the 3 mm Maryland clamp. One reason for missed calibration of the fulcrum point appears that the instruments could only be introduced one cm deep into the thoracic cavity due to the small size of the piglets (Fig. 3).

The esophagus was isolated while sparing the vagal nerve. An approximately 8 mm long segment was resected and the ends anastomosed end to end with interrupted Vicryl 5–0 TF-1 suture.

### Lobectomy of the right upper lobe

The piglet was placed almost prone, the camera port was placed about 5 cm paraspinally to the right in the mid thoracic height. The ports for the 3 mm left and right instruments were placed cranially and caudally to the camera port and more posterior situated for triangulation to the lung. For retraction, an additional port was placed caudally to the left arm port.

The thorax was insufflated with CO_2_ and the piglet was ventilated on both lungs. The right upper lobe, vessels, and the bronchus were identified. The bronchus was cut between clips (Hem-o-Lok®), the vessels divided between sutured ligations (5–0 Vicryl TF-1). The upper lobe was mobilized but not extracted through an incision, as other different procedures were performed in the same piglet.

### Nissen fundoplication (5 procedures)

Nissen fundoplication was performed 5 times, because the restricted operative space in the subdiaphragmatic region with the close proximity of the esophagus to the hepatic veins, the very short intra-abdominal length of the esophagus in piglets and the young liver being extremely vulnerable to grasping and retraction resulted difficult exposition of the esophagus, opening of the right pleural space and in bleeding with termination of one procedure.

The piglet was placed in anti-Trendelenburg’s position. The liver was retracted by a 5 mm blunt grasper or a 10 mm laparoscopic fan retractor (ENDO-Retract 2, Covidien, Germany) inserted through an additional port in the left upper quadrant. The camera port was replaced from the umbilicus to a position 3 cm in the midline above the umbilicus for better access to the subdiaphragmatic region, the left and right hand port were placed in triangulation to the esophagus.

After visualization and dissection of the esophagus, the fundus was mobilized with the robotic 5 mm ultrasound device. Then, a gastric 360° Nissen wrap was placed around the esophagus and stitched with interrupted sutures (3–0 Vicryl SH).

### Hiatoplasty

This procedure was chosen to simulate access to the retroesophageal space as is needed in hiatoplasty or resection of the median arcuate ligament in Dunbar syndrome.

The piglet was placed in anti-Trendelenburg’s position. The liver was retracted by a 5 mm blunt grasper or a 10 mm laparoscopic fan retractor (ENDO-Retract 2, Covidien, Germany) inserted through an additional port in the left upper quadrant. The camera port was placed 3 cm in the midline above the umbilicus for better access to the subdiaphragmatic region, the left and right hand ports were placed in triangulation to the esophagus.

Access to the hiatus was obstructed by the vulnerable liver, careful retraction and dissection exposed the hiatus, which resulted in opening the right hemithroax. Hiatoplasty was performed by interrupted 3–0 Vicryl SH sutures.

### Diaphragmatic plication

Applied to improve lung ventilation in phrenic nerve paralysis, we used this procedure as a model for diaphragmatic surgery. The piglet was placed in anti-Trendelenburg’s position. The liver was retracted by a 5 mm blunt grasper or a 10 mm laparoscopic fan retractor (ENDO-Retract 2, Covidien, Germany) inserted through an additional port in the left upper quadrant (Δ auxiliary port to camera port: 10 cm). The camera port was placed in the umbilicus, the left and right hand ports were placed in triangulation to the esophagus. The left diaphragm was exposed and plicated with interrupted 2–0 Ethibond slipping knots. Because of the size of the sutures, 5 mm robotic instruments were chosen for suturing and knot tying.

### Gastrostomy and gastrostomy closure

For establishing a gastrostomy, the camera port was placed in the umbilicus, the left and right arm ports in triangulation to the gastric body. The anterior wall of the stomach was opened with 3 mm monopolar hook cautery and sutured to a corresponding incision of the abdominal wall. An assistant port in the right upper quadrant was used for retraction and fixation of the stomach onto the visceral peritoneum while suturing (Vicryl 3–0 SH, interrupted stitches). After completion of the gastrostomy, the sutures were taken down and the gastric defect sewn over with interrupted stitches (Vicryl 3–0 SH).

### Gastroenterostomy

This procedure was chosen to simulate intestinal anastomosis and bypass surgery as for obstructing tumors or bariatric reasons. The camera port was placed 1 cm below the umbilicus, the left and right arm ports in triangulation to the gastric greater curvature. The anterior wall of the stomach was opened with monopolar hook cautery. An adjacent intestinal loop was grasped and opened longitudinally with monopolar hook cautery to an according length to the gastric opening. The gastroenterostomy was placed in an isoperistaltic fashion with interrupted sutures (3–0 Vicryl SH).

### Gastro-gastrostomy

This procedure was chosen to simulate duodeno-duodenostomy, as access to and anatomy of the duodenum of piglets did not allow for direct simulation of this procedure. The camera port was placed in the umbilicus, the left and right arm ports in triangulation to the gastric body. The anterior wall of the stomach was incised transversely and a second incision was placed longitudinally caudally from the first. The placement of the incisions resembled the diamond shaped anastomosis in duodenal atresia repair. The gastro-gastrostomy was sutured with interrupted Vicryl 3–0 SH applying two 3 mm needle drivers.

### Entero-enterostomies (2 procedures)

The camera port was placed in the umbilicus, the left and right arm ports in triangulation to the gastric greater curvature. Two loops of small intestine were placed next to each other, incised longitudinally with monopolar hook cautery, and anastomosed with an interrupted or running suture each (5–0 Vicryl TF-1, 10 cm).

### Atypical liver resection

The camera port was placed in the umbilicus, the left and right arm 3 mm ports in triangulation to the left lobes of the liver. The liver of the piglets was extremely vulnerable to grasping and retraction. The most peripheral lobe was visualized and an atypical wedge resection of a representative part of the liver was resected with 3 mm monopolar and 3 mm bipolar hemostasis. Another wedge was resected applying the robotic 5 mm ultrasound device on the right arm. We did not encounter any bleedings or bile leakage.

### Nephroureterectomy (4 procedures, right kidney)

As we were experiencing an accidental iatrogenic laceration of the caval vein with termination of the first we performed three more procedures to examine its feasibility and safety: the piglet was placed in an almost prone to 20° dorsally rotated position. Access to the right kidney was gained with the camera port inserted 2 cm to the right of the umbilicus, the left and right arm were positioned in triangulation to the right kidney. An additional port for retraction of the bowel was inserted caudally to the left arm port.

After visualization of the right kidney, the afferent and efferent vessels and the ureter were isolated. The vessels were separated under 5–0 Vicryl ligatures or 5 mm robotically applied clips (Hem-o-Lok®). A second renal artery with consecutive bleeding and the clip applied for hemostasis tearing the caval vein was the reason for termination of the first procedure. After vascular control the other kidneys were completely mobilized, the ureter suture ligated (5–0 Vicryl) and cut next to its entry into the bladder. The kidney was not extracted through an incision to the outside of the abdominal cavity but placed into the pelvis, as multiple procedures were performed in those piglets.

### Ureteroenterostomy

The ureteroenterostomy was chosen to simulate the suturing of a mobile ureter to intestine in bladder reconstruction. The piglet was placed in an almost prone to 20° dorsally rotated position. Access to the right kidney was gained with the camera port inserted 2 cm to the right of the umbilicus, the left and right arms were positioned in triangulation to the right kidney. The ureter was mobilized and cut; the distal end was suture ligated. The proximal ureter was anastomosed to an opened intestinal loop with 5–0 Vicryl TF-1.

### Pyeloplasty

The piglet was placed in an almost prone to 20° dorsally rotated position. Access to the right kidney was gained with the camera port inserted 2 cm to the right of the umbilicus, the left and right arm were positioned in triangulation to the right kidney. An additional port for retraction of the bowel was inserted caudally to the left arm port. The kidney was exposed, the vessels identified and marked with loops (Vicryl 3–0). The renal pelvis was isolated and cut longitudinally. Transverse reconstruction was done with interrupted 5–0 Vicryl TF-1 sutures.

### Complications and technical limitations

We experienced two bleedings leading to the termination of the live procedures: one was an accidental laceration of the caval vein during nephrectomy, the other an accidental laceration of the hepatic veins during dissection of the esophagus for Nissen fundoplication.

We did not experience any complication related to the robotic system. Although calibration for the fulcrum point resulted in excessive bending in 6 of the 34 procedures, this did not lead to any damage to the animals. Also, the insertion depth of more than 1 cm required for successful calibration did not lead to internal instrument-organ collisions while the system was automatically calibrating and, therefore, moving the instruments inside the animals. The amount of collisions is displayed in Table 1. Instrument—organ and external collisions most often appeared when operating in restricted spaces, but did not lead to injuries of the animals or damage of the robotic arms. We encountered less collisions after replacing the ports to a position with better triangulation to the target, as is also seen in classic laparoscopic surgery. Excessive bending of the instruments was seen during automatic calibration for the fulcrum point in laparoscopic and especially thoracoscopic procedures (Table 1): the ribs of the piglet limited the motion of the instruments resulting in bending which could also be overcome by repositioning of the port or the cart of the robotic arm.

For camera activation and movement by eye tracking we experienced a lag of activation and the movement speed too fast for the small cavities operated in. This led to multiple repositioning movements for the correct camera view or even skipping to manually controlling camera movements using the console clutches.

## Discussion

We were able to successfully perform all planned pediatric surgical procedures with 3 mm robotic instruments in piglets with a median body weight of less than 7 kg.

### Limitations

This study is a single cohort non comparative evaluation of the general feasibility, safety, and technical limitations of a robotic system (Senhance®, Transenterix, USA) in piglets of less than 10 kg, therefore, simulating neonatal and infant robotic assisted laparoscopic surgery. As we did not compare this system to open, laparoscopic or robotic procedures with the Da Vinci system (Intuitive, USA), no conclusions can be drawn concerning any inferiority or superiority of this system to existing pediatric surgical techniques.

The surgical procedures were performed by dedicated pediatric (T.K., R.B.) and general surgeons (J-H. E.) under experimental settings with no time pressure. Any conclusions concerning the application of the robotic system on human cases have to be drawn with caution.

This study is a general test of feasibility and safety of robotic infant surgery with the Senhance. As we did not want to compare surgical techniques, we did not record any scores like the Objective Structured Assessment of Technical Skills (OSATS). After demonstration of its general safety and feasibility, the next step should be a direct comparison of the robot to open and laparoscopic procedures in simulated infants—there the recording and evaluation of specific scores will be helpful.

#### Complications

Although we did not experience any robotic associated complications, two iatrogenic venous lacerations led to elective termination of the live procedures. The first was an accidental laceration of the caval vein during clip-ligating the renal artery undergoing nephrectomy. The other an accidental incision of the hepatic veins, as they run in close proximity to the abdominal esophagus in piglets and both structures are situated directly behind a delicately vulnerable liver lobe, increasing the complexity of careful retraction and dissection in this area.

#### Fulcrum point settings

During the procedures, we encountered technical limitations of the current settings of the system, which have to be addressed: the calibration of the fulcrum point is an automated process the robot is performing by itself by measuring the force exhibited on the instruments while moving them downwards. Therefore, the calibration of the fulcrum point is dependent on the compliance of the abdominal or thoracic wall and the rigidity of the instrument. As the neonatal abdominal wall exhibits a high compliance, calibration for the fulcrum point was unsuccessful in some piglets with certain instruments such as 3 mm graspers whereas 3 mm bipolar instruments appeared to be more rigid, therefore, supporting the calibration. Furthermore, the depth of the instrument insertion for successful calibration appeared to be at least 5–10 cm, a shorter insertion of the instruments resulted in miscalculations and unsuccessful calibration in small cavities. After extensive discussion with the manufacturer, a solution will be to reprogram the algorithm for the calibration with a higher sensitivity adapted to the compliance of the infant abdominal wall without any additional hardware as more sensitive sensors.

A workaround for the situation of repeated unsuccessful calibration is to “simulate the fulcrum point” by manually holding the instrument during calibration at the level the surgeon wants the fulcrum point to be. As the neonatal abdominal or thoracic wall is compliant and thin, even a simulated fulcrum point slightly outside of it will not lead to excessive force or damage. Furthermore, the ability to simulate the fulcrum point is beneficial for surgical procedures such as trans-oral interventions where the instruments are applied in “free air” (manuscript under development).

#### Collisions

Instrument–organ and instrument–instrument collisions appear to increase with decreasing operative space [11]. Therefore, procedures in small cavities such as in neonates and infants are prone to involuntary collisions with potential life threatening complications. A practical instruction would be to place instruments as far from each other as possible, as we were able to demonstrate that increasing the distance from the camera to the left and right hand instrument from 2.5 to 3.5 cm each led to a decrease of collisions from 4 down to 2 during inanimate model suturing drills [11]. Pediatric surgeons should thus “use every inch available” for distanced instrument placement. As this study was conducted to test for the principal feasibility and safety of robotic procedures in simulated infants, we cannot draw any conclusion about optimal placement of the three robotic manipulator arms. These data have to be obtained by a trial comparing open, laparoscopic and robotic procedures.

#### Excessive bending

Excessive bending of the 3 mm instruments was noted in six procedures. One mechanical issue may be the rigidity of the thoracic wall in thoracoscopic procedures, but bending has also been observed in abdominal procedures. Whenever the operating space is constricted, such as in thoracic and renal procedures, bending occurred but could be reduced by repositioning of the arms of the robot. Undocking the robot, repositioning the cart and arms then re-docking the system is a time consuming task that can be sped up or avoided completely by strategically placing the ports and the cart and arms of the robot before the begin of the procedure: Excessive bending could be reduced by placing the instrument and the manipulator arm in a straight line pointing towards the designated target area. This requires a relevant space (footprint) for all three arms around the operating table, which may be constrained in certain operating theaters. Sometimes, excessive bending was noted after miscalculations of the fulcrum point. It is thus essential for the surgeon to directly visualize and control the fulcrum point calibration and repeat it if there is doubt of the correct fulcrum point settings. Undocking the arm, repositioning and recalibrating the fulcrum point during the procedure to relief bending is far more time consuming.

#### Camera movement

Camera movement is initiated and directed by eye tracking, which is calibrated to each user at the setup of the user specific account. Up/down and left/right movement of the camera is initiated by looking at the specific area the surgeon wants to move the camera to. The zoom in/zoom out function, which triggers physical camera insertion or extraction, is initiated by the surgeon moving his head forward and backward. We experienced a delay of the initiation of camera movement and, after the delay, a speed of movement that appeared to be too fast, especially in small cavities as the thorax of a 6 kg piglet. Although we did not experience any collision of the camera with internal organs or structures, as we learned to anticipate this effect of “lag and speed”, the general lag and consecutive speed of camera movement during the zoom in/zoom out function is a critical point for surgery in newborns.

By holding daily technical conferences with the manufacturer of the system during the course of the study, this issue was addressed: The zoom in/zoom out function was reprogrammed to include a delay and increase the speed of movements after multiple feedback of adult general surgeons in prior evaluations. Therefore, reprogramming the system with no delay and slower movement appears to be a solution.

### Advantages and disadvantages of the Senhance compared to the da Vinci system

Comparing the Senhance to the da Vinci, we experienced the following advantages and disadvantages based on our study and clinical applications: The main advantages of the Senhance are the 3 mm diameter instruments which lead to a reduction in operative space with probable application in newborns and infants. The specific disadvantage is that those 3 mm instruments are not wristed, the surgeon has, therefore, no improved manual dexterity compared to traditional 3 mm instrument laparoscopy except tremor filtering and movement scaling. The da Vinci offers 5 mm wristed instruments but due to their size of the 5 mm angulating tips they require relevant more operative space than their 8 mm counterparts and still more than 3 mm instruments (a volume of 216 ml versus 91 ml) [5, 7, 11]. The development of 3 mm wristed instruments with short articulated tips will, therefore, improve robotic newborn and infant surgery.

An advantage of the Senhance is the lesser distance required between the ports for the instruments compared to the da Vinci, which can be as short as 2.5 cm and is thus better suitable for small abdominal cavities of infants [11].

Both systems offer a 3D stereoscopic vision, although the closed console of the da Vinci appears to improve “immersion” of the surgeon into the operative field similar to microscopy compared to the open console with stereoscopic glasses of the Senhance. The Senhance’ camera movement is controlled by eye tracking, to activate the user has to trigger two specific buttons, each on one handle, which is similar in the da Vinci. Whether the actual steering of the camera by eye tracking of the Senhance or movement of the manipulator handles of the da Vinci is superior to the other appears to be a matter of personal preference. We initially experienced a lag of Senhance camera movement upon activation of eye tracking as well as the speed of the camera movement too fast for the small cavities of the neonatal piglets. Reprogramming the camera movement by the manufacturer was discussed. Alternatively, the camera movement can be controlled by hand in the Senhance similar to the da Vinci.

Tactile force feedback, offered only by the Senhance, may improve safety compared to the da Vinci, but we were not able to palpate or feel organs or tissue in the newborn piglets and did not encounter any interruption of instrument movements upon collision with internal organs, which were rare (Table 1). Therefore, tactile force feedback, with its current sensitivity settings of the Senhance, does not appear be advantageous in newborn robotic surgery.

### Comparison to literature results of infant robotic surgery

Unfortunately, there are no current data on robotic surgery of infants with the Senhance and not much data concerning the da Vinci system. A series of 100 da Vinci robotic cases included 22 children with a weight of less than 10 kg.[15, 16] Any specific considerations for this age group were not given in this report. While there are several more series reported of da Vinci robotic surgery in children, comprehensive data on infants are rare [14, 15]. In one report on pediatric urology, the ability to perform robotic surgery was restricted by collisions when infants (mean weight 7.99 ± 1.03 kg) had a distance between both anterior and superior iliac spines of 13 cm or less or a puboxyphoid distance of 15 cm or less [8]. In another report, robotic assisted pyeloplasty was performed in three children with a weight between 5 and 8 kg.[16] Meehan reported his experience with 47 robotic procedures in children less than 10 kg. He reported to apply a 5 mm 2D camera for thoracic procedures—which loose one advantage of the robot (3D vision). The only robotic esophageal repair refistulized 2 weeks after and had to be revised by open thoracotomy. 10% of cases had to be converted to either open or laparoscopic surgery. One recommendation given was similar to our results as to place the robotic instruments as far apart as possible, even compared to laparoscopy [14]. Based on those small case series, robotic da Vinci surgery appears not to be critically harmful to infants, although data on collisions and complications relating to different age and weight groups are still missing. Whether a general recommendation should be given for robotic infant surgery based on the available case series has to be discussed: we propose a three step approach of evaluation of new robotic systems for application in infants (1. inanimate model, 2. live animal model, 3. comparison of open, laparoscopic and robotic procedures in live animal models).

### Robotic surgery in infants

Neonatal and infant surgery is highly specialized and needs thorough surgical and ethical scrutiny. Therefore, one could argue, that any application of robotic systems in neonates or infants has to be proven equivalent safe and effective to open or laparoscopic procedures in comparative animal studies before applying this devices in newborns without reliable data. This is of upmost importance, as randomized prospective data on oncological gynecologic surgery demonstrated a negative effect of minimally invasive laparoscopic or robotic surgery on patient survival compared to open surgery in early stage uterine cervical carcinoma [17, 18].

Currently, the Senhance has FDA clearance and CE Mark approval for surgery in children with a body weight of more than 10 kg. Any surgery in infants of less than 10 kg would be, therefore, off-label, the same with the da Vinci system. We were able to demonstrate that the Senhance can be operated in small cavities without any robot associated complications in piglets of 5–7 kg body weight. Although it, therefore, appears safe to apply the Senhance in infants within this weight category, more qualified data have to be complied before general consideration of its application in infants. We deem our study being the second of the three steps of the evaluation of surgical robots for application in infants as described above: the first step is the assessment of the robot in an inanimate model simulating small cavities such as in infants [11]. After passing step 1, the second step is the evaluation of the robot in a live model simulating infants (this report). The third step should be the comparison of open, laparoscopic and robotic procedures in live models simulating infants. Only after passing all three steps, a recommendation for or against of the use of a specific robot in infants should be concluded.

For practical clinical application, we currently do not consider either the da Vinci or Senhance to perform robotic surgery in infants: the da Vinci appears not to have passed our suggested step 1 as we know from inanimate studies that it requires an operative space with at least a virtual cube of 6 × 6 × 6 cm (216 ml) [5, 7, 8]. For the Senhance the step 3 trial is under development.

Nevertheless, surgeon and patient driven application of robotic surgery leads to off-label use before systematic data can be obtained. Procedures with the da Vinci in newborns and infants have been reported in uncontrolled case series [13, 14].

## Conclusion

Robotic newborn and infant surgery appears technically feasible with the 3 mm and 5 mm instruments. We have been able to successfully complete 34 procedures without any robotic associated complications.

The camera movement was faster than desired in the small cavities, but has the potential to be optimized via software updates to the robotic system as well as the modification of the calibration for the fulcrum point setting to a higher sensitivity to adjust for the compliance of the abdominal wall of newborns and infants. Further evaluation of the Senhance system is needed via prospective experimental and then clinical trials comparing it to open, laparoscopic and other robotic technologies.
